# A new set-valued system identification approach to identifying rare genetic variants for ordered categorical phenotype

**DOI:** 10.1186/1471-2105-15-S10-P29

**Published:** 2014-09-29

**Authors:** Wenjian Bi, Guolian Kang, Yuehua Cui, Yun Li, Christine M Hartford, Wing Leung, Ji-Feng Zhang

**Affiliations:** 1 Key Laboratory of Systems and Control, Academy of Mathematics and Systems Science, Chinese Academy of Sciences, Beijing 100190, PRC; 2 Department of Biostatistics, St. Jude Children’s Research Hospital, Memphis, TN 38105, USA; 3 Department of Statistics and Probability, Michigan State University, East Lansing, MI 48824, USA; 4 Department of Biostatistics, University of North Carolina, 3101 McGavran-Greenberg Hall, Chapel Hill, NC 27599, USA; 5 Department of Bone Marrow Transplantation and Cellular Therapy, St. Jude Children’s Research Hospital, Memphis, TN 38105, USA; 6 Department of Pediatrics, University of Tennessee Health Science Center, Memphis, TN, 38163, USA

## Background

For phenotype-genotype association studies that involve a phenotype with ordered multiple response categories, we usually either regroup multiple categories of the phenotype into two categories of “cases” and “controls” and then apply the standard logistic regression (LG) model [1,2], or apply a non-parametric method of Spearman rank correlation [3] or parametric method of ordered logistic (orderLG) regression model [4] which accounts for the ordinal nature of the phenotype. However, these approaches may lose statistical power if the phenotype is obtained by categorizing an observed or complicated unmeasured or immeasurable continuous phenotype or if the underlying genetic variants are rare.

## Materials and methods

Therefore, we propose a set-valued (SV) system method, which assumes that the underlying continuous phenotype follows a normal distribution, to identify genetic variants associated with an ordinal categorical phenotype. We couple this model with a set-valued system identification method to identify all underlying key system parameters.

## Results

Simulation studies show that SV well controlled the Type I error rate. In the comparison among LG, SV and orderLG methods, LG had significantly lower power than both SV and orderLG due to the disregard of the ordinal nature of the phenotype, and SV had similar or higher power than orderLG. Additionally, the SV association parameter estimate was 2.7-28.7 fold less variable than the orderLG association parameter estimate. Less variability in the association parameter estimate translates to greater power and robustness across the spectrum of minor allele frequencies. These advantages are most pronounced for rare variants or even common variants when sample size is small. For instance, in a simulation with data generated from an additive orderedLG model with an odds ratio of 7.4 for a phenotype with three categories, a single nucleotide polymorphism with minor allele frequency of 0.75% and sample size of 999 (333 per category), the power of SV, orderLG and LG models were 70%, 40% and <1%, respectively, at a significance level of 10^-6^. When applied to a real data set, the set of variants identified by LG and orderLG was a subset of those identified by SV. Thus, SV can be a competitive alternative to LG or orderLG in genetic association studies such as candidate gene, genome-wide association studies or next generation sequencing studies, for ordered categorical phenotype.
